# Assessing the Accuracy of Reporting of Hypertension on Death Certificates in Australia

**DOI:** 10.1093/ajh/hpae108

**Published:** 2024-08-13

**Authors:** Tim Adair, Hang Li, Chalapati Rao

**Affiliations:** Nossal Institute for Global Health, Melbourne School of Population and Global Health, The University of Melbourne, Carlton, VIC, Australia; Nossal Institute for Global Health, Melbourne School of Population and Global Health, The University of Melbourne, Carlton, VIC, Australia; National Centre for Epidemiology and Population Health, The Australian National University, Canberra, ACT, Australia

**Keywords:** blood pressure, data linkage, death certificates, hypertension, mortality

## Abstract

**BACKGROUND:**

There is insufficient evidence of how accurately hypertension is reported on death certificates, which are the primary evidence of causes of death. This study assesses the accuracy of reporting of hypertension on death certificates of decedents in Australia who previously had their blood pressure measured.

**METHODS:**

Blood pressure data from the 2014–2015 and 2017–2018 National Health Surveys were linked to death registration data from July 2015 to December 2021 (average 3.3 years from survey to death). The percentage of decedents with hypertension reported on the death certificate was calculated according to blood pressure level and previous diagnosis of hypertension.

**RESULTS:**

Hypertension was reported on the death certificate of 20.2% (95% confidence interval 12.1%–28.3%) of decedents who had very high to severe blood pressure (160/100 mm Hg and above), 14.5% (10.3%–18.8%) who had high blood pressure (140 to <160 / 90 to <100 mm Hg), 14.1% (10.8%–17.4%) who had normal to high blood pressure (<140/90 mm Hg) and who took hypertension medication, and 17.8% (13.6%–22.0%) who had been diagnosed with hypertension. Where the decedent had very high to severe blood pressure, hypertension was reported for 27.9% (14.1%–41.8%) of deaths if they had been diagnosed with hypertension, and 21.7% (9.6%–33.7%) where another cardiovascular disease was reported on the death certificate.

**CONCLUSIONS:**

Hypertension mortality in Australia is only reported for a minority of deaths of people with high or very high to severe blood pressure; this is also found for those with a prior diagnosis of hypertension.

## Graphical Abstract

**Graphical Abstract:**
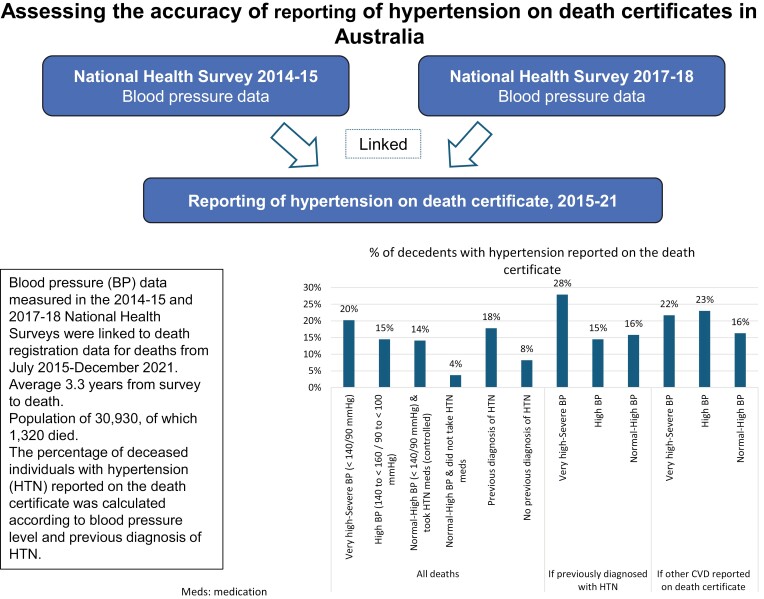

Hypertension, or high blood pressure, is the leading risk factor for mortality globally and in Australia.^1,2^ Optimal control of high blood pressure is essential to ensure improved longevity of the population in the future given the slowdown in the long-term decline in cardiovascular disease mortality in Australia and other high-income countries.^3^

Despite the population health importance of hypertension, there is uncertainty about the precise magnitude of its contribution to mortality. It is unknown how accurately hypertension is reported on the death certificate, or International Form of Medical Certificate of Cause of Death (MCCD), which is the primary source of information on causes of death. The death certificate reports the diseases that led to the person dying, from the underlying cause to the direct cause in a pathophysiological sequence on part 1, and other significant conditions contributing to the death but not related to the underlying cause in part 2.^4^ It is generally believed that hypertension is underreported on death certificates due to it being perceived as a risk factor, rather than as a cause of death. Underreporting of hypertension means that death certificates, in their current form, may not be used to assess the contribution of high blood pressure to mortality. However, death certificates that accurately report hypertension can directly measure the role of the condition in mortality. This contrasts with another method to measure the contribution of hypertension to mortality by burden of disease studies, the population attributable fraction, which is calculated from the disparate sources of population prevalence of high blood pressure in health surveys and relative risks of cause-specific mortality from elevated systolic blood pressure derived from epidemiological studies.^5^

In a review conducted by the authors, the only study that has assessed the accuracy of the reporting of hypertension on the death certificate was conducted in 5 hospitals in Kuwait over 3 decades ago, where hypertension as an underlying cause of death was underreported by 31%.^6^ This lack of evidence about the accuracy of the reporting of hypertension on death certificates is a major impediment to understanding its contribution to mortality. To fill this knowledge gap, this study aims to assess the accuracy of reporting of hypertension on death certificates of deceased individuals in Australia who previously had their blood pressure measured in a national survey.

## METHODS

Blood pressure data were obtained from the 2014–2015 and 2017–2018 National Health Surveys (NHSs).^7,8^ The NHS is a nationally representative sample survey of the health of the Australian population. Blood pressure was measured using an automated blood pressure monitor and was imputed for nonresponses (24% in the 2014–2015 NHS and 32% in the 2017–2018 NHS; see Supplementary Materials online); respondents could refuse to have their blood pressure measured for reasons including their health, concerns about privacy and sensitivity (e.g., culture and gender).^7,8^ We classified measured blood pressure as (i) very high to severe (160/100 mm Hg and above, 6.1% of respondents), (ii) high (140 to <160 / 90 to <100 mm Hg, 16.8%), (iii) normal to high (<140/90 mm Hg) and the respondent reported that they took hypertension medication (see Supplementary Material online) in the 2 weeks before the survey (10.8%, also called “controlled”), and (iv) normal to high but did not take hypertension medication (66.3%). The NHS also includes data on whether the respondent had ever been told by a doctor or nurse that they have high blood pressure/hypertension (19.0% of all respondents).

The study uses death registration data in Australia for deaths occurring from July 2015 to December 2021, a time period commencing after the completion of the 2014–2015 NHS, that allows linkage with the NHS if survey respondents later died.^9^ Hypertension was identified as being reported on the death certificate if it was in either part 1 or 2 and its International Classification of Diseases, 10th Revision (ICD-10) code was from I10 to I15. This grouping includes all types of hypertension.

The death registration data were linked to the NHS via a Personal Linkage Spine in the Person Level Integrated Data Asset (PLIDA) of the Australian Bureau of Statistics (ABS).^10^ In total, the population from the NHS was 30,930 aged 18 years and over, of which 1,320 or 4% died. The average length of time from survey to death was 3.3 years.

Our primary analyses were to assess, of NHS respondents who subsequently died, the percentage who had hypertension reported on the death certificate according to their blood pressure level and reported previous diagnosis of hypertension. For a sensitivity analysis, we restricted our calculations to deaths where another cardiovascular disease (ICD-10 codes I00–I99, excluding hypertension I10–I15 which is our condition of interest) was reported on the death certificate, a situation where blood pressure would more likely contribute to a death compared with other deaths. We calculated 95% confidence intervals of results using the replicate weights technique.^8^ We conducted logistic regression to identify whether blood pressure and previous diagnosis of hypertension were statistically significant predictors of reporting of hypertension on the death certificate, controlling for covariates of age at death, sex of deceased, time interval from survey to death (because a longer time interval may result in the blood pressure measurement being a less reliable predictor or reporting of the condition on the death certificate), and the area-level Index of Relative Socio-economic Disadvantage (IRSD) quintile measured at the Statistical Area Level 1 (SA1, average population 400).^11,12^ Further information about the NHS, death registration, and PLIDA are in the Supplementary Material online. Basic descriptive data, including the leading underlying cause of death according to blood pressure level, are shown in Supplementary Table S1 online.

All data analyses were conducted on the ABS DataLab, where PLIDA data can be accessed. All outputs were approved by the ABS to comply with their rules that each table cell must have a minimum of 10 cases. This meant that categories in some tables could not be disaggregated, such as normal to high blood pressure for most analyses.

Ethics approval was obtained from the University of Melbourne Medicine and Dentistry Human Ethics Sub-Committee (Reference 2023-25166-43934-3). The data underlying this article cannot be shared publicly because they can only be accessed by approved users of PLIDA via the ABS DataLab.

## RESULTS

Table 1 shows that only 20.2% (95% confidence interval 12.1%–28.3%) of decedents who had very high to severe blood pressure (160/100 mm Hg and above) had hypertension reported on the death certificate. This is compared with 14.5% (10.3%–18.8%) of decedents who had high blood pressure (140 to <160 / 90 to <100 mm Hg), 14.1% (10.8%–17.4%) who had normal to high blood pressure (<140/90 mm Hg) and who took hypertension medication (i.e., controlled), and 3.7% who did not take hypertension medication. Multivariate analysis shows that the odds ratio (OR) of very high to severe blood pressure was 3.76 (1.86–7.62), high blood pressure 3.48 (1.79–6.77), and normal to high (controlled) 3.13 (1.61–6.08). Of those who had a previous diagnosis of hypertension, 17.8% (13.6%–22.0%) had hypertension reported on the death certificate compared with 8.2% (5.6%–10.8%) if it had not been diagnosed; the OR was 1.62 (1.14–2.30).

**Table 1.** Whether hypertension^a^ reported on death certificate (2015–2021) by blood pressure and whether previously diagnosed with hypertension (NHS 2014–2015 and 2017–2018), both sexes, 18 years and above

	Reported on death certificate	
	%	OR (95% CI)
1. All deaths (*n* = 1,320)		
Blood pressure		
Normal–high (<140/90 mm Hg)	9.7 (7.5–12.0)	^c^
Did not take HTN medication	3.7 (1.1–6.3)	1
Took HTN medication (controlled)	14.1 (10.8–17.4)	3.13^**^ (1.61–6.08)
High (140 to <160 / 90 to <100 mm Hg)	14.5 (10.3–18.8)	3.48^**^ (1.79–6.77)
Very high–severe (160/100 mm Hg or higher)	20.2 (12.1–28.3)	3.76^**^ (1.86–7.62)
Reported previous diagnosis of hypertension		
Not diagnosed	8.2 (5.6–10.8)	1
Diagnosed	17.8 (13.6–22.0)	1.62^**^ (1.14–2.30)
2. If reported previous diagnosis of hypertension (*n* = 608)		
Blood pressure		
Normal–high^b^	15.8 (11.5–20.2)	1
High	14.5 (7.5–21.4)	0.94 (0.57–1.54)
Very high–severe	27.9 (14.1–41.8)	1.27 (0.72–2.24)
3. If no reported previous diagnosis of hypertension (*n* = 712)		
Blood pressure		
Normal–high^b^	5.1 (2.1–8.2)	1
High	14.6 (7.7–21.6)	2.81^**^ (1.56–5.07)
Very high–severe	10.6 (3.6–17.5)	2.16^*^ (1.05–4.44)

Percentage figures are weighted. Blood pressure includes both measured and imputed. A separate logistic regression was conducted for each of the 3 numbered groups. Each regression also included covariates of age, sex, time interval from survey to death, and area-level Index of Relative Socio-economic Disadvantage (IRSD). Full regression results are shown in Supplementary Tables S3–S5 online. Abbreviations: ABS, Australian Bureau of Statistics; CI, confidence interval; HTN, hypertension; ICD-10, International Classification of Diseases, 10th Revision; *n*, number of cases; NHS, National Health Survey; OR, odds ratio.

^*^ *P* < 0.05.

^**^ *P* < 0.01.

^a^ICD-10 codes I10–I15.

^b^Normal–high blood pressure was unable to be disaggregated by whether they had taken hypertension medication because the number of cases was too small according to ABS disclosure rules.

^c^Normal–high blood pressure was disaggregated into 2 categories (whether or not take HTN medication) in the regression. The reference group in the regression was normal–high blood pressure and did not take hypertension medication.

Restricting the analysis to decedents with a previous diagnosis of hypertension, 27.9% (14.1%–41.8%) of those with very high to severe blood pressure had hypertension reported on the death certificate, compared with 14.5% (7.5%–21.4%) who had high blood pressure and 15.8% (11.5%–20.2%) who had normal–high blood pressure. However, according to the multivariate analysis, there was no statistically significant difference between very high to severe and normal to high (OR 1.27, 0.72–2.24). If there was no reported previous diagnosis of hypertension, both very high to severe (10.6%, 3.6%–17.5%; OR 2.81, 1.56–5.07) and high (14.6%, 7.7%–21.6%; OR 2.81, 1.56–5.07) blood pressure had a higher level of reporting of hypertension on the death certificate than normal to high blood pressure (5.1%, 2.1%–8.2%).

In the sensitivity analysis, the proportion of decedents with hypertension reported where another cardiovascular disease was reported on the death certificate was 21.7% (9.6%–33.7%) for very high to severe, 23.0% (15.7%–30.3%) for high, and 16.3% (11.6%–21.1%) for normal to high blood pressure; according to multivariate analysis, there was no statistically significant difference between these figures (Supplementary Table S2 online). Those with a previous diagnosis of hypertension had a higher level of reporting of the condition on the death certificate (24.7%, 18.0%–31.4%) than those who did not (9.5%, 4.9%–14.2%), with an OR of 2.12 (1.41–3.18). However, where another cardiovascular disease was not reported on the death certificate, there was a large difference between decedents who had very high to severe blood pressure (18.0%, 5.6%–30.4%; OR 2.84, 1.29–6.24) and those who had high (4.8%, 1.4%–8.1%) and normal–high (4.1%, 1.8%–6.4%) blood pressure.

## DISCUSSION

This study’s findings demonstrate that hypertension mortality in Australia is significantly underreported. Hypertension was reported on the death certificate of only one-fifth of decedents who had very high to severe blood pressure, one-seventh who had high blood pressure, and also one-seventh of those with controlled normal–high blood pressure. Even if another cardiovascular disease was reported on the death certificate, where it would be more likely that hypertension would be a cause of death, this figure was less than one-quarter.

One reason for the low reporting of hypertension on the death certificate is that it is underdiagnosed; in the 2014–2015 and 2017–2018 NHSs, only 43% of people in Australia with very high to severe and 33% with high blood pressure have been diagnosed with the condition.^7,8^ Significant underdiagnosis of people with hypertension has been found globally because it can often present without any obvious symptoms and can only be detected through specific examination using instruments; hence it is referred to as the “Silent Killer.”^13,14^

While having a previous diagnosis of hypertension increased reporting of the condition on the death certificate, for people with very high to severe blood pressure and diagnosed hypertension it was reported on <30% of death certificates. Although hypertension may not be regarded by physicians as the underlying cause or a direct cause of death in part 1 of the death certificate, it may be more suitable to be reported as another significant condition contributing to the death in part 2. Qualitative research among physicians would help identify their attitudes toward and understanding of the clinical and public health importance of hypertension and the reporting of the condition on the death certificate.

A limitation of the study is that blood pressure was measured on average 3.3 years before death, although it is unclear what impact this had; it is possible that some people with high blood pressure subsequently had the condition controlled with hypertension medication that was not reported in the NHS, while others who were undiagnosed with the condition at the time of the survey were later diagnosed. Blood pressure was also only measured on a single occasion, which raises the possibility that the reading was incorrect because clinical practice guidelines recommend average measures over multiple visits to a clinic. Our analyses included blood pressure data that, for between one-quarter and one-third of cases depending on the survey, was imputed by the ABS for nonresponse (see Supplementary Material online for imputation method); ABS data show that blood pressure statistics for measured and imputed cases are almost identical to measured cases only.^7,8^ Another limitation is that hypertension would not contribute to all deaths of people with high blood pressure, however, even in the clearest example of it having a contribution (very high–severe, diagnosed, another cardiovascular disease reported), it was only reported on 25% of death certificates.

This study used a unique linked dataset to assess the accuracy of reporting of hypertension on death certificates, the first study in recent decades to do so. It raises major concerns about how accurately hypertension’s contribution to mortality is being measured and in Australia it suggests that current estimates that high blood pressure contributes to 11%–15% of all deaths, whether based on the burden of disease studies or calculated directly from death certificates, are too low.^15–17^ These findings have relevance for other high-income countries, such as the United States, that face similar challenges from the burden of hypertension. Higher estimates of mortality from high blood pressure, as demonstrated by this study, would significantly revise estimates of its burden of disease to prioritize health interventions and allocate resources to reduce cardiovascular disease mortality risk.^18–20^ Further data from cohort studies and clinical trials for which details on cause-specific mortality as well as antecedent morbidity would be available could be used to refine such estimates of the burden of disease from hypertension. Importantly, improved reporting of hypertension on death certificates could be achieved through appropriate technical guidance to physicians on the importance of good cause of death certification practices.

## Supplementary Material

hpae108_suppl_Supplementary_Tables
