# Supplementary material for: Assessing the Accuracy of Reporting of Hypertension on Death Certificates in Australia
Source: Am J Hypertens. 2024 Aug 13;37(12):948–52. doi: 10.1093/ajh/hpae108 (PMC11565191; doi:10.1093/ajh/hpae108)
Supplement: hpae108_suppl_Supplementary_Tables [file hpae108_suppl_supplementary_tables.docx]

**Assessing the accuracy of reporting of hypertension on death certificates in Australia**

**Supplementary material**

**Further information on data sources**

The 2014-15 National Health Survey (NHS) was conducted from July 2014 to June 2015 and collected data from 19,259 respondents, while the 2017-18 NHS was conducted from July 2017 to June 2018 and collected data from 21,315 respondents ^1,2^. Both systolic and diastolic pressures of respondents 18 years and over were measured by the interviewer using an automated blood pressure monitor. The voluntary nature of these measurements meant that there was non-response of 24% in the 2014-15 NHS and 32% in the 2017-18 NHS ^1,2^. Respondents are able to refuse to have their blood pressure measured for reasons including their health, concerns about privacy and sensitivity (e.g. culture and gender).^1^ The Australian Bureau of Statistics (ABS) impute blood pressure for missing values in the NHS using the ‘hot decking’ imputation method, whereby a case with a missing response is assigned the response of a case with similar characteristics; the characteristics used were age, sex, part of state (capital city and balance of state), self-perceived body mass (underweight, acceptable, or overweight), level of exercise, high cholesterol as a long-term condition and self-reported body mass index (from reported height and weight) ^1^. Our analyses used both measured and imputed blood pressure to increase the number of cases in our analyses so that they meet the ABS minimum cell count requirements. The ABS state that measured blood pressure data in the 2014-15 and 2017-18 NHSs are directly comparable ^2^.

The NHS also contains data of what medication had been taken in the two weeks before the survey. For hypertension medication, we included antihypertensives, diuretics, beta blocking agents, calcium channel blockers and agents acting on the renin-angiotensin system; this classification was used by the Australian Institute of Health and Welfare in their reporting of blood pressure from the NHS ^3^.

Deaths in Australia are registered by each state and territory’s Registry of Births, Deaths and Marriages and compiled by the ABS. Death registration data comprise all deaths registered in Australia and include all diseases and conditions reported in Part 1 and Part 2 of the death certificate. The death certificate is completed by a certifier (normally the attending physician) and the diseases and conditions are classified by the International Classification of Diseases, 10^th^ Revision (ICD-10), which is the global standard for measuring cause-specific mortality. ICD-10 classifies hypertensive diseases as essential (primary) hypertension (ICD-10 code I10), hypertensive heart disease (I11), hypertensive chronic kidney disease (I12), hypertensive heart and chronic kidney disease (I13) and secondary hypertension (I15) ^4^.

The NHS also includes data of the ABS Index of Relative Socio-economic Disadvantage (IRSD), which summarizes the economic and social conditions of people and households within an area. It only includes measures of relative disadvantage such as low income, low education, unemployment, long-term health condition or disability, overcrowding of dwelling and being unable to speak English well.^5^

The ABS Person Level Integrated Data Asset (PLIDA) aims to identify all individuals residing in Australia at any point in time based on Medicare, Centrelink and Income Tax data ^6^. Linkage rates with the Personal Linkage Spine are 98.5% of deaths from July 2015-December 2021 in the death registration dataset, 94.9% of 2014-15 NHS cases and 91.7% of 2017-18 NHS cases.

**References**

1. Australian Bureau of Statistics. 4363.0 - National Health Survey: Users' Guide, 2014-15 Australian Bureau of Statistics: Canberra, 2017.

2. Australian Bureau of Statistics. 4363.0 - National Health Survey: Users' Guide, 2017-18 ABS: Canberra, 2019.

3. Australian Institute of Health and Welfare. High blood pressure. Australian Institute of Health and Welfare: Canberra, 2019.

4. World Health Organization. International Statistical Classification of Diseases and Related Health Problems 10th Revision (ICD-10): 2016 Version. World Health Organization: Geneva, 2016.

5. Australian Bureau of Statistics. 2033.0.55.001 - Census of Population and Housing: Socio-Economic Indexes for Areas (SEIFA), Australia, 2016. Australian Bureau of Statistics: Canberra, 2018.

6. Australian Bureau of Statistics. Personal linkage spine. Australian Bureau of Statistics: Canberra, 2023.

**Table S1: Blood pressure, previous diagnosis of hypertension, age, sex (NHS 2014-15 and 2017-18) and underlying cause of death according to blood pressure level of decedents (2015-21), both sexes, 18 years and above**

| **Blood pressure** |  |
| --- | --- |
| Normal-High (Less than 140/90 mmHg) |  |
| & did not take HTN medication | 66.3% |
| & took HTN medication (controlled) | 10.8% |
| High (140 to less than 160 / 90 to less than 100 mmHg) | 16.8% |
| Very high-Severe (160/100 mmHg or higher) | 6.1% |
| **Previous diagnosis of HTN** |  |
| Diagnosed | 46.5% |
| Not diagnosed | 53.6% |
| **Age (years)** |  |
| Mean | 73.7 |
| Median | 77 |
| 25^th^ percentile | 66 |
| 75^th^ percentile | 84 |
| **Sex** |  |
| Male | 56.0% |
| Female | 44.0% |
| **Leading non-communicable disease underlying causes of death** |  |
| ***Normal-high (controlled), high, very high-severe blood pressure*** |  |
| Cancers | 31.2% |
| Cardiovascular diseases | 29.9% |
| Respiratory diseases | 7.7% |
| Endocrine, nutritional and metabolic diseases | 5.7% |
| Diseases of the digestive system | 4.8% |
| ***Normal-high (not controlled) blood pressure*** |  |
| Cancers | 44.0% |
| Cardiovascular diseases | 19.0% |
| Respiratory diseases | 9.0% |
| Diseases of the nervous system | 4.1% |
| Mental and behavioral disorders | 3.1% |

Figures are weighted. Blood pressure includes both measured and imputed.

**Table S2: Whether hypertension^+^ reported on death certificate (2015-21) by blood pressure and whether previously diagnosed with hypertension (NHS 2014-15 and 2017-18), where other cardiovascular disease reported on the death certificate, both sexes, 18 years and above**

|  | **Reported on death certificate** | |
| --- | --- | --- |
|  | **%** | **OR (95% CI)** |
| 1. ***If other CVD^$^ reported on death certificate (n=672)*** | | |
| **Blood pressure** | | |
| Normal-High^¶^ (Less than 140/90 mmHg) | 16.3 (11.6-21.1) | 1 |
| High (140 to less than 160 / 90 to less than 100 mmHg) | 23.0 (15.7-30.3) | 1.47 (0.94-2.30) |
| Very high-Severe (160/100 mmHg or higher) | 21.7 (9.6-33.7) | 1.07 (0.62-1.83) |
| **Reported previous diagnosis of hypertension** | | |
| Not diagnosed | 9.5 (4.9-14.2) | 1 |
| Diagnosed | 24.7 (18.0-31.4) | 2.12** (1.41-3.18) |
|  | | |
| 1. ***If other CVD^$^ not reported on death certificate (n=648)*** | | |
| **Blood pressure** | | |
| Normal-High^¶^ | 4.1 (1.8-6.4) | 1 |
| High | 4.8 (1.4-8.1) | 1.33 (0.62-2.82) |
| Very high-Severe | 18.0 (5.6-30.4) | 2.84* (1.29-6.24) |
| **Reported previous diagnosis of hypertension** | | |
| Not diagnosed | 3.6 (1.5-5.7) | 1 |
| Diagnosed | 13.6 (8.6-18.6) | 1.58 (0.83-3.01) |

Percentage figures are weighted. Blood pressure includes both measured and imputed. A separate logistic regression was conducted for each of the five numbered groups. Each regression also included covariates of age, sex, time interval from survey to death and area-level Index of Relative Socio-economic Disadvantage (IRSD). Full regression results are shown in Tables S6-S7 in the Supplementary Material.

*p<0.05 **p<0.01

**^+^** ICD-10 codes I10-I15 ^$^ ICD-10 codes I01-I09, I20-I99.

^¶^ Normal-high blood pressure unable to be disaggregated by whether they had taken hypertension medication because number of cases too small according to ABS disclosure rules.

n: Number of cases. CI: Confidence interval. NHS: National Health Survey. OR: Odds ratio. HTN: Hypertension. CVD: Cardiovascular disease.

**Table S3: Logistic regression of whether hypertension reported on the death certificate (2015-21), both sexes, 18 years and above**

| **Variable** | **Odds ratio (95% confidence interval)** |
| --- | --- |
| **Blood pressure^** (Ref. Normal-High (Less than 140/90 mmHg) & did not take HTN medication) |  |
| Normal-High & took HTN medication (controlled) | 3.13** (1.61-6.08) |
| High (140 to less than 160 / 90 to less than 100 mmHg) | 3.48** (1.79-6.77) |
| Very high-Severe (160/100 mmHg or higher) | 3.76** (1.86-7.62) |
| **Previous diagnosis HTN** (Ref. Not diagnosed) |  |
| Diagnosed | 1.62** (1.14-2.30) |
| **Age (years)** | - 1. (1.00-1.03) |
| **Sex** (Ref. Male) |  |
| Female | 1.35 (0.97-1.88) |
| **Time interval from survey to death (years)** | 1.07 (0.98-1.17) |
| **IRSD quintile** (Ref. 1: lowest) |  |
| 2 | 0.78 (0.49-1.24) |
| 3 | 0.93 (0.58-1.49) |
| 4 | 1.21 (0.74-1.98) |
| 5: highest | 0.73 (0.41-1.29) |
| **Constant** | 0.01** (0.00-0.02) |

N=1,320 *p<0.05 **p<0.01

^ Measured and imputed blood pressure.

HTN: Hypertension. IRSD: Index of Relative Socio-economic Disadvantage.

**Table S4: Logistic regression of whether hypertension reported on the death certificate (2015-21), if reported previous diagnosis of hypertension, both sexes, 18 years and above**

| **Variable** | **Odds ratio (95% confidence interval)** |
| --- | --- |
| **Blood pressure^** (Ref. Normal-High (Less than 140/90 mmHg)) |  |
| High (140 to less than 160 / 90 to less than 100 mmHg) | 0.94 (0.57-1.54) |
| Very high-Severe (160/100 mmHg or higher) | 1.27 (0.72-2.24) |
| **Age (years)** | 1.02 (1.00-1.04) |
| **Sex** (Ref. Male) |  |
| Female | 1.60* (1.04-2.45) |
| **Time interval from survey to death (years)** | 1.08 (0.97-1.20) |
| **IRSD quintile** (Ref. 1: lowest) |  |
| 2 | 0.90 (0.50-1.63) |
| 3 | 1.16 (0.62-2.16) |
| 4-5 | 1.14 (0.66-1.98) |
| **Constant** | 0.02** (0.00-0.10) |

N=602 *p<0.05 **p<0.01

^ Measured and imputed blood pressure.

HTN: Hypertension. IRSD: Index of Relative Socio-economic Disadvantage.

Note: IRSD quintiles 4 and 5 combined to meet ABS data output rules.

**Table S5: Logistic regression of whether hypertension reported on the death certificate (2015-21), if no reported previous diagnosis of hypertension, both sexes, 18 years and above**

| **Variable** | **Odds ratio (95% confidence interval)** |
| --- | --- |
| **Blood pressure^** (Ref. Normal-High (Less than 140/90 mmHg)) |  |
| High (140 to less than 160 / 90 to less than 100 mmHg) | 2.81** (1.56-5.07) |
| Very high-Severe (160/100 mmHg or higher) | 2.16* (1.05-4.44) |
| **Age (years)** | 1.02* (1.00-1.05) |
| **Sex** (Ref. Male) |  |
| Female | 1.05 (0.62-1.78) |
| **Time interval from survey to death (years)** | 1.07 (0.93-1.25) |
| **IRSD quintile** (Ref. 1: lowest) |  |
| 2 | 0.55 (0.26-1.18) |
| 3 | 0.64 (0.31-1.33) |
| 4-5 | 0.67 (0.34-1.33) |
| **Constant** | 0.01** (0.00-0.07) |

N=703 *p<0.05 **p<0.01

^ Measured and imputed blood pressure.

HTN: Hypertension. IRSD: Index of Relative Socio-economic Disadvantage.

Note: IRSD quintiles 4 and 5 combined to meet ABS data output rules.

**Table S6: Logistic regression of whether hypertension reported on the death certificate (2015-21), if other CVD^$^ reported on death certificate, both sexes, 18 years and above**

| **Variable** | **Odds ratio (95% confidence interval)** |
| --- | --- |
| **Blood pressure^** (Ref. Normal-High (Less than 140/90 mmHg)) |  |
| High (140 to less than 160 / 90 to less than 100 mmHg) | 1.47 (0.94-2.30) |
| Very high-Severe (160/100 mmHg or higher) | 1.07 (0.62-1.83) |
| **Previous diagnosis HTN** (Ref. Not diagnosed) |  |
| Diagnosed | 2.12** (1.41-3.18) |
| **Age (years)** | 1.01 (0.99-1.03) |
| **Sex** (Ref. Male) |  |
| Female | 1.45 (0.97-2.16) |
| **Time interval from survey to death (years)** | 1.01 (0.91-1.13) |
| **IRSD quintile** (Ref. 1: lowest) |  |
| 2 | 0.86 (0.50-1.49) |
| 3 | 1.05 (0.59-1.86) |
| 4 | 1.07 (0.58-1.99) |
| 5: highest | 0.86 (0.45-1.65) |
| **Constant** | 0.04** (0.01-0.19) |

N=672 *p<0.05 **p<0.01 ^$^ ICD-10 codes I01-I09, I20-I99.

^ Measured and imputed blood pressure.

HTN: Hypertension. IRSD: Index of Relative Socio-economic Disadvantage. CVD: Cardiovascular disease

**Table S7: Logistic regression of whether hypertension reported on the death certificate (2015-21), if other CVD^$^ not reported on death certificate, both sexes, 18 years and above**

| **Variable** | **Odds ratio (95% confidence interval)** |
| --- | --- |
| **Blood pressure^** (Ref. Normal-High (Less than 140/90 mmHg)) |  |
| High (140 to less than 160 / 90 to less than 100 mmHg) | 1.33 (0.62-2.82) |
| Very high-Severe (160/100 mmHg or higher) | 2.84* (1.29-6.24) |
| **Previous diagnosis HTN** (Ref. Not diagnosed) |  |
| Diagnosed | 1.58 (0.83-3.01) |
| **Age (years)** | 1.02 (0.99-1.05) |
| **Sex** (Ref. Male) |  |
| Female | 1.54 (0.81-2.91) |
| **Time interval from survey to death (years)** | 1.17 (0.99-1.38) |
| **IRSD quintile** (Ref. 1: lowest) |  |
| 2-3 | 0.68 (0.32-1.44) |
| 4-5 | 1.01 (0.45-2.26) |
| **Constant** | 0.00** (0.00-0.03) |

N=648 *p<0.05 **p<0.01 ^$^ ICD-10 codes I01-I09, I20-I99.

^ Measured and imputed blood pressure.

HTN: Hypertension. IRSD: Index of Relative Socio-economic Disadvantage. CVD: Cardiovascular disease

Note: IRSD quintiles 2 to 3 and 4 to 5 combined to meet ABS data output rules.
